# Supplementary material for: Cryo-EM structure of amyloid fibrils formed by the entire low complexity domain of TDP-43
Source: Nat Commun. 2021 Mar 12;12:1620. doi: 10.1038/s41467-021-21912-y (PMC7955110; doi:10.1038/s41467-021-21912-y)
Supplement: Supplementary file 1 — Supplementary Information [file 41467_2021_21912_MOESM1_ESM.pdf]

Supplementary Information

Cryo-EM structure of amyloid fibrils formed by the entire low complexity domain of TDP-43

Qiuye Li, W. Michael Babinchak, Witold K. Surewicz

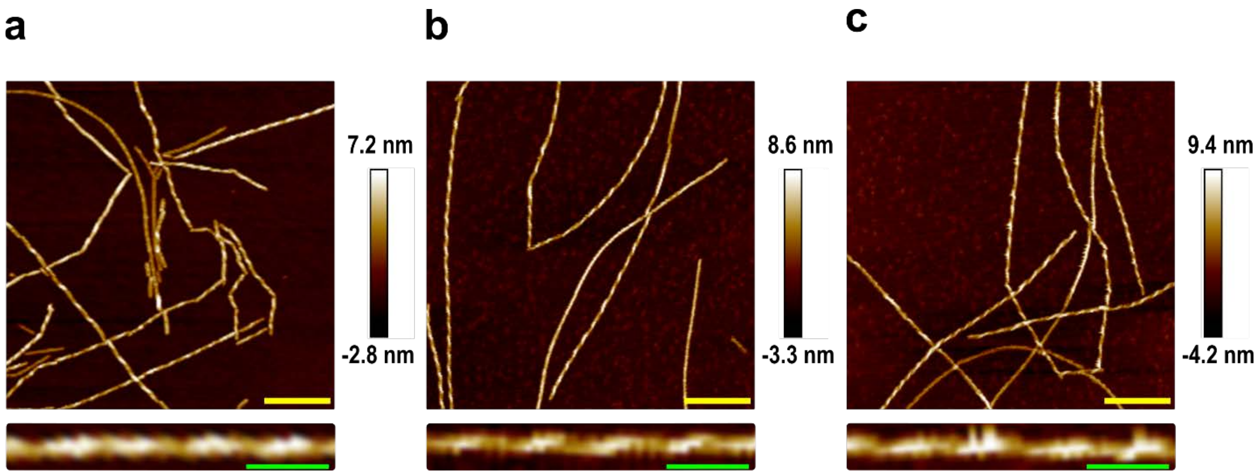

Supplementary Figure. 1. AFM images of TDP-43 LCD fibrils spontaneously formed at pH 6 (a) and pH 4 (b), as well as last generation seeded fibrils formed at pH 4 (c). Enlarged images of representative twisted fibrils are shown in the bottom panels. All fibrils are left-handed with a similar height and periodicity (see text for details). The same fibril morphologies were observed in 20 images for each type of sample. Yellow scale bars correspond to 200 nm; green scale bars correspond to 50 nm.

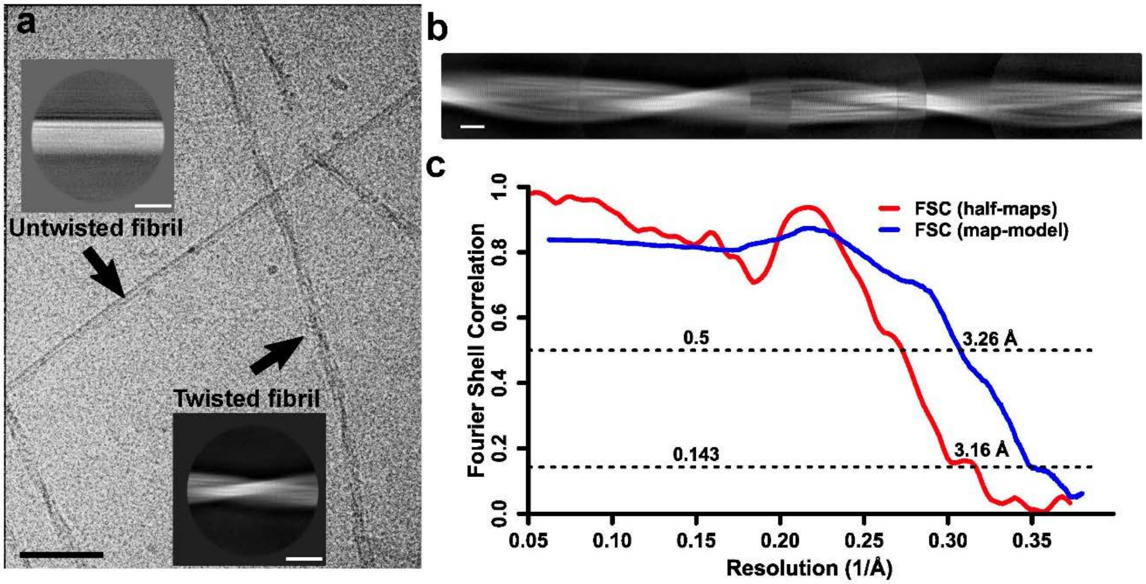

Supplementary Figure. 2. Cryo-EM data processing. a, Two different types of morphologies found for TDP-43 LCD fibrils. Cryo-EM images show both twisted and untwisted fibrils, the same fibril morphologies were observed in at least 4,000 images; scale bar (black) corresponds to 50 nm. 2D class averages of each fibril type are shown as insets; scale bars (white) correspond to 10 nm. **b,** Manually assembled full pitch of TDP-43 LCD fibrils from multiple 2D class averages, scale bar corresponds to 10 nm. **c**, Fourier shell correlation curves between the two independently refined half-maps (red) and between the map and the atomic model (blue).

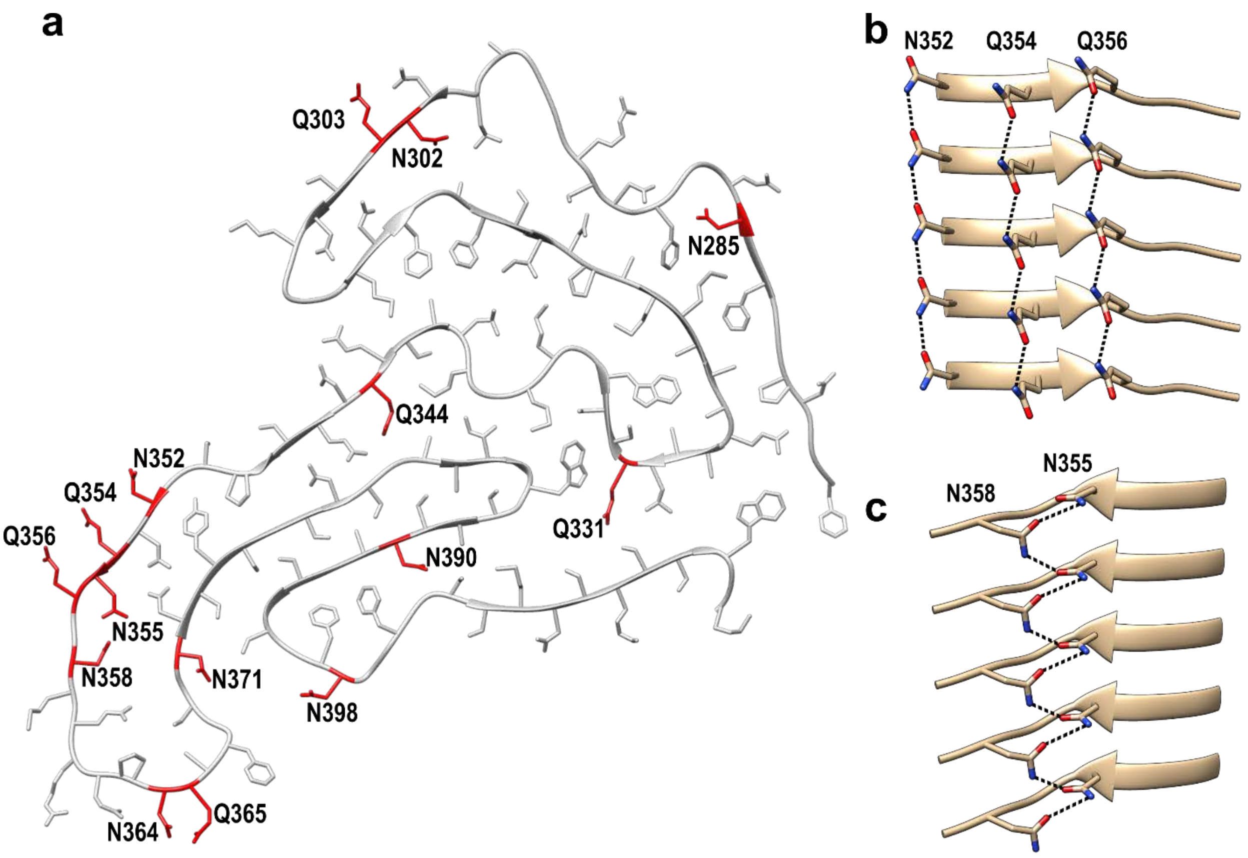

Supplementary Figure. 3. Illustration of stabilizing interlayer hydrogen bonds between stacked Asn and Gln side chains. a, Top view showing Asn and Gln residues (marked in red) involved in interlayer hydrogen bonding. **b**, Asn/Gln ladders of Asn352, Gln354, and Gln356 with hydrogen bonds (black dashed lines) between the same residues in adjacent subunits. **c**, Ladders of Asn355 and Asn358 in which hydrogen bonds are between different residues in adjacent subunits.

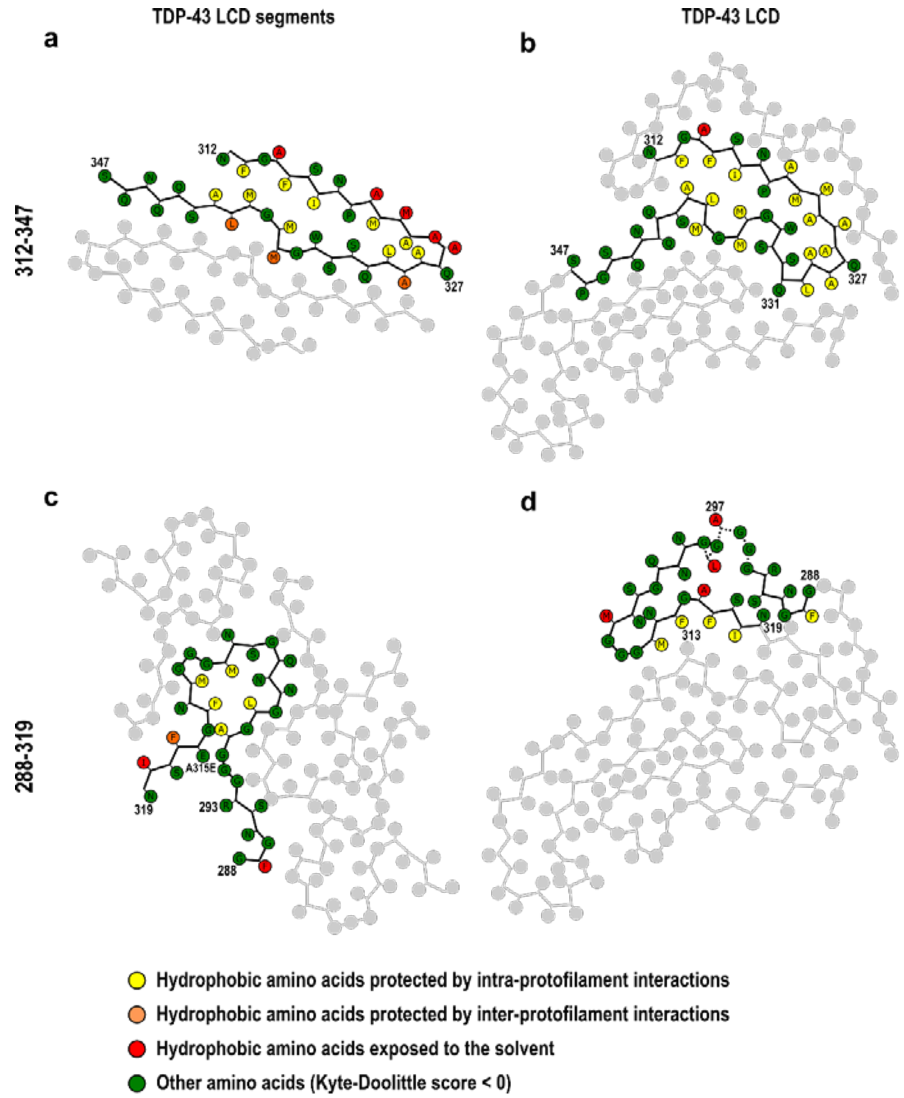

Supplementary Figure. 4. Comparison of structural motifs in fibrils generated from relatively short fragments of TDP-43 LCD[1] with those in the same regions of fibrils generated from the entire LCD. **a**, Representative structure of fibrils formed by the 311-360 fragment (PDB 6N37). Almost half of hydrophobic residues within the dagger motif (residues 312-347) are either exposed to water or protected by intermolecular interactions with another protofilament (depicted in gray). **b**, The structure of this region within a much larger amyloid core of fibrils formed by the entire LCD is substantially different. This region is buried in the interior of a single protofilament, with almost all hydrophobic residues involved in intra-protofilament interactions and protected from water (see also Fig. 1d). **c**, The structure of the inner protofilament in fibrils formed by the 286-331 fragment with A315E mutation showing an R-shaped fold within the 288-319 segment, with a salt bridge between Arg293 and Asp315 (PDB 6N3C). Three other protofilaments seen in the structure are shown in gray. A similar R-shaped fold was proposed to be adopted by fibrils formed by this fragment in the absence of any mutation, with the structure stabilized by hydrophobic interactions between Ala297, Phe313, and Ala315. **d**, The structure within the 288-319 region of fibrils formed from the entire LCD is quite different.

Supplementary Table 1. Cryo-EM data collection, refinement, and validation statistics

**Data collection and processing**	
Magnification	× 105,000
Voltage (kV)	300
Electron dose (e^-/Å^2)	42
Defocus range (μm)	-0.8 to -1.5
Pixel size (Å)	0.828
Symmetry imposed	C1
Initial particles images (no.)	294,168
Final particle images (no.)	11,026
Map Resolution (Å)	3.16
FSC threshold	0.143
**Refinement**	
Initial model used	De novo
Map sharpening B factor (Å^2)	-29.83
Model composition	
Non-hydrogen atoms	4,695
Protein residues	695
B factors (Å^2)	
protein	94.85
R.M.S.D	
Bond lengths (Å)	0.006
Bond angles (˚)	1.170
MolProbity score	2.07
Clashscore	12.59
Poor rotamers (%)	0
Ramachandran plot	
Favored (%)	92.70
Allowed (%)	7.30
Disallowed (%)	0

Supplementary References

1. Cao, Q., Boyer, D. R., Sawaya, M. R., Ge, P. & Eisenberg, D. S. Cryo-EM structures of four polymorphic TDP-43 amyloid cores. *Nat. Struct. Mol. Biol.* **26**, 619–627 (2019).
