# Supplementary material for: Cryo-EM structure of amyloid fibrils formed by the entire low complexity domain of TDP-43
Source: Nat Commun. 2021 Mar 12;12:1620. doi: 10.1038/s41467-021-21912-y (PMC7955110; doi:10.1038/s41467-021-21912-y)
Supplement: Supplementary file 3 — Reporting Summary [file 41467_2021_21912_MOESM3_ESM.pdf]

nature research

Corresponding author(s): Witold K. Surewicz

Last updated by author(s): Feb 15, 2021

Reporting Summary

Nature Research wishes to improve the reproducibility of the work that we publish. This form provides structure for consistency and transparency in reporting. For further information on Nature Research policies, see our Editorial Policies and the Editorial Policy Checklist.

Statistics

For all statistical analyses, confirm that the following items are present in the figure legend, table legend, main text, or Methods section.

n/a	Confirmed	
☐	☒	The exact sample size (n) for each experimental group/condition, given as a discrete number and unit of measurement
☐	☒	A statement on whether measurements were taken from distinct samples or whether the same sample was measured repeatedly
☒	☐	The statistical test(s) used AND whether they are one- or two-sided<br>*Only common tests should be described solely by name; describe more complex techniques in the Methods section.*
☒	☐	A description of all covariates tested
☒	☐	A description of any assumptions or corrections, such as tests of normality and adjustment for multiple comparisons
☒	☐	A full description of the statistical parameters including central tendency (e.g. means) or other basic estimates (e.g. regression coefficient) AND variation (e.g. standard deviation) or associated estimates of uncertainty (e.g. confidence intervals)
☒	☐	For null hypothesis testing, the test statistic (e.g. F, t, r) with confidence intervals, effect sizes, degrees of freedom and P value noted<br>*Give P values as exact values whenever suitable.*
☒	☐	For Bayesian analysis, information on the choice of priors and Markov chain Monte Carlo settings
☒	☐	For hierarchical and complex designs, identification of the appropriate level for tests and full reporting of outcomes
☒	☐	Estimates of effect sizes (e.g. Cohen's d, Pearson's r), indicating how they were calculated

Our web collection on statistics for biologists contains articles on many of the points above.

Software and code

Policy information about availability of computer code

Data collection	AFM images were collected by NanoScope 9.1. Automated cryo-EM data collection was driven by SerialEM 3.7.14.
Data analysis	AFM images were processed by NanoScope Analysis 1.5. Cryo-EM data were processed by RELION 3.1, MotionCor2 1.2.2, Gctf 1.06. The atomic model was built and refined by Coot 0.8.9.2 and PHENIX 1.18. Steric clashes of atomic models were determined by Reduce 3.23 and Probe 2.16. Figures of density map and atomic model were prepared in UCSF Chimera 1.14.

For manuscripts utilizing custom algorithms or software that are central to the research but not yet described in published literature, software must be made available to editors and reviewers. We strongly encourage code deposition in a community repository (e.g. GitHub). See the Nature Research guidelines for submitting code & software for further information.

Data

Policy information about availability of data

All manuscripts must include a data availability statement. This statement should provide the following information, where applicable:

- Accession codes, unique identifiers, or web links for publicly available datasets
- A list of figures that have associated raw data
- A description of any restrictions on data availability

Cryo-EM density map is available through Electron Microscopy Data Bank (EMDB) with ID EMD-23059. The atomic model is available through Protein Data Bank (PDB) with ID 7KWZ.

Field-specific reporting

Please select the one below that is the best fit for your research. If you are not sure, read the appropriate sections before making your selection.

☒ Life sciences ☐ Behavioural & social sciences ☐ Ecological, evolutionary & environmental sciences

For a reference copy of the document with all sections, see nature.com/documents/nr-reporting-summary-flat.pdf

Life sciences study design

All studies must disclose on these points even when the disclosure is negative.

Sample size	The reported height maxima and periodicity of twisted fibrils were based on measurements using twenty randomly selected fibrils. To access the percentage of twisted fibrils, the total length of twisted and non-twisted fibrils in three randomly selected 2 μm × 2 μm AFM images was measured. Each image contains at least 50 fibrils. Similar sample sizes were used in the literature for similar studies [e.g., Lin et al. 2019 (doi.org/10.1021/acs.jpcb.9b07854); Lee et al. 2020 (doi.org/10.1038/s41467-020-19512-3)].
Data exclusions	In cryo-EM data analysis, 294,168 particles were extracted from the raw data and those that did not contribute to a high-resolution 3D density map were discarded. 11,026 particles were used for the final reconstruction.
Replication	Identical pH 4 and pH 6 fibril morphologies were obtained in three independent sample preparations.
Randomization	Randomization was used only for the Fourier-shell correlation analysis in Supplementary Fig. 2c. Randomization serves no purpose in other analysis.
Blinding	Blinding was not used because this is a study of molecular structure. No subjective assessments of group or categories were involved. Blinding would serve no purpose.

Reporting for specific materials, systems and methods

We require information from authors about some types of materials, experimental systems and methods used in many studies. Here, indicate whether each material, system or method listed is relevant to your study. If you are not sure if a list item applies to your research, read the appropriate section before selecting a response.

Materials & experimental systems

n/a	Involved in the study	
☒	☐	Antibodies
☒	☐	Eukaryotic cell lines
☒	☐	Palaeontology and archaeology
☒	☐	Animals and other organisms
☒	☐	Human research participants
☒	☐	Clinical data
☒	☐	Dual use research of concern

Methods

n/a	Involved in the study	
☒	☐	ChIP-seq
☒	☐	Flow cytometry
☒	☐	MRI-based neuroimaging
